# Enriched Mechanical Strength and Bone Mineralisation of Electrospun Biomimetic Scaffold Laden with Ylang Ylang Oil and Zinc Nitrate for Bone Tissue Engineering

**DOI:** 10.3390/polym11081323

**Published:** 2019-08-08

**Authors:** Mohan Prasath Mani, Saravana Kumar Jaganathan, Eko Supriyanto

**Affiliations:** 1 School of Biomedical Engineering and Health Sciences, Faculty of Engineering, Universiti Teknologi Malaysia, Skudai 81310, Malaysia; 2 Department for Management of Science and Technology Development, Ton Duc Thang University, Ho Chi Minh City, Vietnam; 3 Faculty of Applied Sciences, Ton Duc Thang University, Ho Chi Minh City, Vietnam; 4 IJNUTM Cardiovascular Engineering center, School of Biomedical Engineering and Health Sciences, Faculty of Engineering, Universiti Teknologi Malaysia, Skudai 81310, Malaysia

**Keywords:** polyurethane, ylang ylang/zinc nitrate, physico-chemical properties, calcium deposition, bone tissue engineering

## Abstract

Scaffolds supplemented with naturally derived materials seem to be a good choice in bone tissue engineering. This study aims to develop polyurethane (PU) nanofibers added with ylang ylang (YY) and zinc nitrate (ZnNO_3_) using the electrospinning method. Field emission scanning electron microscopy (FESEM) images showed that the diameter of the PU nanofibers (869 ± 122 nm) was reduced with the addition of YY and ZnNO_3_ (PU/YY—467 ± 132 nm and PU/YY/ZnNO_3_—290 ± 163 nm). Fourier transform infrared (FTIR), a thermal gravimetric analysis (TGA) and an X-ray diffraction (XRD) analysis confirmed the interactions between PU with YY and ZnNO_3_. In addition, a thermal gravimetric analysis (TGA) study revealed the improved thermal stability for PU/YY and a slight reduction in the thermal stability for PU/YY/ZnNO_3_. A tensile test indicated that the addition of YY and ZnNO3 (PU/YY—12.32 MPa and PU/YY/ZnNO_3_—14.90 MPa) improved the mechanical properties of the pristine PU (6.83 MPa). The electrospun PU/YY (524 nm) and PU/YY/ZnNO_3_ (284 nm) showed a reduced surface roughness when compared with the pristine PU (776 nm) as depicted in the atomic force microscopy (AFM) analysis. The addition of YY and ZnNO_3_ improved the anticoagulant and biocompatibility nature of the pristine PU. Furthermore, the bone mineralization study depicted the improved calcium deposition in the fabricated composites (PU/YY—7.919% and PU/YY/ZnNO_3_—10.150%) compared to the pristine PU (5.323%). Hence, the developed composites with desirable physico-chemical properties, biocompatibility and calcium deposition can serve as plausible candidates for bone tissue engineering.

## 1. Introduction

In bone tissue engineering, artificial bone scaffolds are used to support the remodeling of the bone defects. The bone defects were caused by disease or trauma, which include tumour ablation, bone cysts, osteolysis, and neurosurgical defects [1]. Bone regeneration is a complex process involving the interaction of different cell types such as chondrocytes, mesenchymal stem cells, osteoblasts, osteoclasts and endothelial cells [2]. In bone tissue engineering, the scaffolds play a vital role in carrying the cells and also for cellular ingrowth. An ideal scaffold for the bone substitute should be able to resemble the biological and mechanical properties of natural bone tissue [3]. Polymeric scaffold ranging from natural to synthetic has received huge attention in bone tissue engineering, owing to their biocompatibility and biodegradability [4]. Bone tissue engineering is comprised of three components, namely (1) cells (2) scaffolds and (3) growth factors. The scaffold, an important component, is a three-dimensional (3D) structure that can support cell adhesion and proliferation for new tissue growth [5]. Furthermore, the artificial scaffolds should possess a biomimetic structure which could have the ability to mimic the native function of the extracellular matrix (ECM) [6,7]. An artificial ECM-based scaffold shows many advantages like (1) providing the mechanical support required for the cell growth, (2) having the ability to control cell functions (3), and offering an appropriate environment for facilitating the cell attachment [3]. In this study, an artificial ECM-mimicking scaffold was fabricated by electrospinning.

Electrospinning is a cost-effective and versatile technique that generates a fibrous scaffold with a diameter ranging from the micrometre to the nanometre range [8]. The fibrous scaffold possesses a high surface area with a porous structure that can resemble the native ECM structure and is widely used in biomedical applications [9,10]. Fibrous scaffolds were reported to enhance the cell adhesion, migration and proliferation [11]. Furthermore, they also provide a superior environment for cellular ingrowth to non-fibrous scaffolds. In this research, polyurethane (PU) is selected for fabricating the scaffold, which is a poly ether-based polymer. PU is selected because it has several advantages, like biocompatibility, biodegradability and a good oxidation stability [12,13]. Furthermore, the electrospun scaffolds based on PU were widely used in wound dressing, tissue engineering and drug delivery systems [14,15,16].

In this research, the polyurethane was added with additives to improve the cellular non-toxic behaviour and also to enhance the mechanical strength, in order to support the new bone tissue. Recently, it was observed that the electrospun scaffolds, added with natural products like essential oils, honey and propolis, have enhanced the cellular response [17,18,19]. Furthermore, a few studies have reported that the addition of metallic particles into the electrospun scaffold have improved the tensile strength [20,21]. Hence, this study aims to fabricate PU blended with a combination of essential oil and metallic particles as a scaffold for bone tissue engineering.

In this research, ylang ylang (YY) oil and Zinc nitrate (ZnNO_3_) were utilized as additives for PU scaffolds. *Cananga odorata* is known as ylang-ylang (YY), which is a fast-growing tree widely found in the tropical areas of Asia such as Malaysia, Philippines, India, Indonesia and the islands of the Indian Ocean. Ylang-ylang essential oil contains monoterpene hydrocarbons, sesquiterpene hydrocarbons, oxygen-containing monoterpenes, oxygen-containing sesquiterpenes, acetates, benzenoids, benzoates and phenols. The oxygenated fraction of YY essential oil contains the main active components of p–methylanisole, methyl benzoate, benzyl benzoate, benzyl acetate, geranyl acetate, cinnamyl acetate, (E,E)–farnesyl acetate, linalool, geraniol, and benzyl salicylate. Meanwhile, the main active components from the hydrocarbon fraction of ylang-ylang oil are germacrene D, β–caryophyllene, γ–Muurolene and (E,E)–farnesyl acetate. *C. odorata* is reported to have medicinal properties and is traditionally used for treating various diseases like malaria, asthma pneumonia and stomach ache. Certain studies had reported that the bioactive extracted from the YY was reported to possess biomedical properties like antibacterial, antioxidant, antipest, anti-inflammatory and antifungal activities. [22]. ZnNO_3_ was utilized to improve the mechanical strength of the electrospun scaffold. It has been observed that the scaffold containing zinc particles exhibited a non-toxicity to the osteoprogenitor cells and enhanced the antibacterial activity. In addition to the antibacterial properties, the zinc is reported to support the osteoblast activity, apatite formation and enhanced adhesion and proliferation of osteoblast cells [23]. This study aims to electrospun and test the bone scaffold based on polyurethane added with YY and ZnNO_3_.

## 2. Experimental Section

### 2.1. Materials

Tecoflex EG 80A, medical grade aliphatic polyurethane, was purchased from Lubrizol, Wickliffe, OH, United States. The solvent dimethylformamide (DMF) utilized for dissolving the polymer was supplied from Merck, Burlington, NJ, USA. YY oil was purchased from AEON, Johor, Malaysia. Phosphate buffer saline (PBS) and sodium chloride physiological saline (0.9% *w*/*v*) were obtained from Sigma-Aldrich, Kuala Lumpur, Malaysia. ZnNO_3_ was supplied from Sigma Aldrich, Gillingham, UK. The clotting reagents used for the coagulation study were obtained from Diagnostic Enterprise, Thiruvananthapuram, India.

### 2.2. Preparation of PU and Their Composite Solution

The weight concentration of PU used was 9%, and it was obtained by dissolving 0.450 g in 5 mL of DMF. Similarly, the homogeneous solution of YY (4 v/v%) and ZnNO_3_ (4 wt %) was prepared by adding 120 µL and 0.120 g of YY and ZnNO_3_ in 3 mL of DMF. Finally, the prepared YY and ZnNO_3_ was added to the PU homogenous solution to make PU/YY and PU/YY/ZnNO_3_ at a volume ratio of 8:1 v/v% and 8:0.5:0.5 v/v% respectively.

### 2.3. Electrospinning of Prepared Solutions

The electrospinning equipment (Progene Link Sdn Bhd, Selangor, Malaysia) was used to convert the prepared solutions into the fibrous scaffold, and this was done at room temperature with a humidity of 55%. The operation parameters were kept constant for electrospinning of all of the prepared solutions. The optimized voltage, flow rate and collector distance were 11.5 kV, 0.3 mL/h and 20 cm, respectively. The obtained fibers that were collected used aluminium foil placed on the collector drum. The fibers were dried under vacuum to remove any residues that were present.

### 2.4. Characterization

#### 2.4.1. Field Emission Scanning Electron Microscopy (FESEM)

The morphological analysis of the fibrous membrane was done using the FESEM unit (Hitachi SU8020, Tokyo, Japan). Prior to imaging, membranes were sputtered with gold and imaged for different magnifications. The mean fiber diameter of the electrospun membranes was determined using Image J by selecting 30 locations from the particular captured image.

#### 2.4.2. Fourier Transform Infrared Spectroscopy (FTIR)

The infrared spectrum of the electrospun membranes was done using Nicolet iS 5, Thermo Fischer Scientific, Waltham, MA, USA. The ATR crystal that was used was zinc selenium. For each sample, the 32 scans were performed in the wavelength range of 600–4000 cm^−1^ at a resolution of 4 cm^−1^.

#### 2.4.3. Contact Angle Measurements

The water contact angle, which depicts the wettability of the fabricated membranes, was measured by video contact angle (VCA) unit (AST products, Inc., Billerica, MA, USA). Drops of deionized water (0.5 μL) were applied on the electrospun surfaces, and using high resolution camera a static image was captured within a few seconds. The contact angles for the static image were calculated using computer integrated software, and the experiment was repeated three times.

#### 2.4.4. X-ray Diffraction

An X-ray diffractometer analyser (Rigaku, Tokyo, Japan) with CuKα radiation was used to analyse the crystallographic changes in the electrospun membranes. Patterns were attained in the 2θ range of 10–90° with a scanning rate of 10°/min.

#### 2.4.5. Thermal Behaviour

The thermal behaviour of PU and their composite was determined using a thermal gravimetric analysis (TGA) unit (PerkinElmer, Waltham, MA, USA). The thermal behaviour was studied between the temperature ranges of 30 to 1000 °C with a heating rate of 10 °C/min under a nitrogen atmosphere.

#### 2.4.6. Tensile Test Analysis

The mechanical performance of the electrospun membranes was determined by a uniaxial tester machine (Gotech Testing Machines, AI-3000, Taichung City, Taiwan). The samples with a size of 40 × 15 mm^2^ were cut and clamped in the grips of the machine with a gauge length of 20 mm. The experiment was performed at a cross-head speed of 10 mm/min with a load cell of 500 N. The average tensile strength was determined from the machine plotted stress-strain curve.

#### 2.4.7. Atomic Force Microscopy (AFM) Analysis

The surface topography of the nanofibrous scaffolds was evaluated by an atomic force microscopy unit (NanoWizard^®^, JPK Instruments, Berlin, Germany) in taping mode. The experiment was performed in a normal atmosphere and the scanning area of 20 × 20 µm^2^ with a 256-sample resolution.

### 2.5. Mineralization Testing

The mineralization test was done using simulated body fluid (SBF) to determine the bioactivity of the electrospun membranes. A small cut sample of the electrospun membranes was soaked in 1.5x SBF (pH 7.4; 37 °C) and incubated for 14 days. The soaked samples were removed, and after 14 days they were cleaned with distilled water. After this, the samples were dried at 37 °C. The dried electrospun membranes were examined through scanning electron microscopy (SEM) unit (Hitachi Tabletop TM3000, Tokyo, Japan) equipped with Energy Dispersive X-Ray Spectroscopy (EDX) to determine the amount of calcium deposition.

### 2.6. Coagulation Assays

#### 2.6.1. Activated Partial Thromboplastin Time (APTT) Assay

Electrospun PU and its composites scaffolds were cut into square samples and incubated in PBS at 37 °C for 30 min before starting the assay. First, the samples were placed with 50 µL of obtained PPP for 1 min at 37 °C, followed by adding 50 µL of rabbit brain cephaloplastin reagent for 3 min at 37 °C. Finally, the mixture was activated by adding 50 µL of CaCl_2_ and stirred using a sterile steel needle. The time taken for the clot formation was noted using a chronometer.

#### 2.6.2. Prothrombin Time (PT) Assay

Electrospun PU and its composites scaffolds were cut into square samples and incubated in PBS for 30 min at 37 °C. To begin the assay, the samples were placed with 50 µL of PPP for 1 min, followed by adding 50 µL of NaCl–thromboplastin reagent (Factor III) and stirring using a sterile steel needle. The time taken for the formation of clot was noted as PT.

#### 2.6.3. Hemolysis Assay

To start this assay, both PU and its composites scaffolds were soaked in physiological saline (0.9% w/v) at 37 °C for 30 min. Then, they were exposed to the mixture of citrated blood and diluted saline (4:5) for 1 h at 37 °C. After this, the exposed samples were taken out, and the mixtures were centrifuged at 3000 rpm for 15 min. Finally, the supernatant was aspirated, and the absorbance was recorded at 542 nm, which represents the damage of red blood cell (RBCs). The hemolytic percentage was calculated using the following formula [18]:

Hemolysis percentage (HP) = (TS − NC)/(PC − NC) × 100(1)

where TS, NC, and PC are the measured absorbance values of the test sample, negative control, and positive control at 542 nm, respectively.

### 2.7. MTS Assay

An MTS (3-(4,5-dimethylthiazol-2-yl)-5-(3-carboxymethoxyphenyl)-2-(4-sulfophenyl)-2H-tetrazolium) assay was used to determine the cell growth levels on electrospun scaffolds after a 5 day culture. A small cut piece of electrospun scaffolds was placed into 96 well plates and sterilized with 70% ethanol for 10 min and cleansed with PBS. After this, human dermal fibroblast (HDF) cells were seeded into each well, in which scaffolds were placed with a density of 10 × 10^3^ cells/cm^2^ and cultured for 5 days. After 5 days, the scaffolds were removed from the medium and further incubated with 20% of MTS at 37 °C for four hours. The live cells in the electrospun membranes were measured by taking the absorbance at 490 nm using a spectrophotometer reader. The experiments were done in triplicate.

### 2.8. Statistical Analysis

All of the experiments were done thrice independently. A one-way ANOVA was performed to calculate the statistical significance (*p* < 0.05) and was expressed as the mean ± SD. In the case of the qualitative experiments, an illustration of three images is indicated.

## 3. Results and Discussion

### 3.1. SEM Investigation

**Figure 1:**
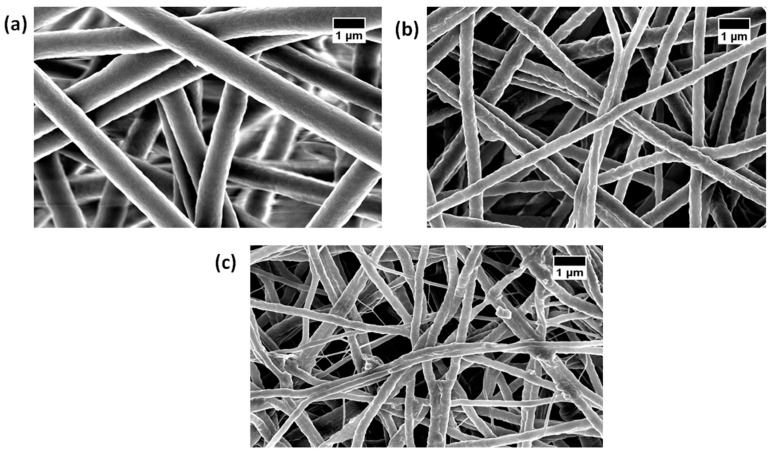
Field Emission Scanning Electron Microscopy (FESEM) images of (**a**) polyurethane (PU), (**b**) PU/ylang ylang (YY) and (**c**) PU/YY/zinc nitrate (ZnNO_3_)_._

**Figure 2:**
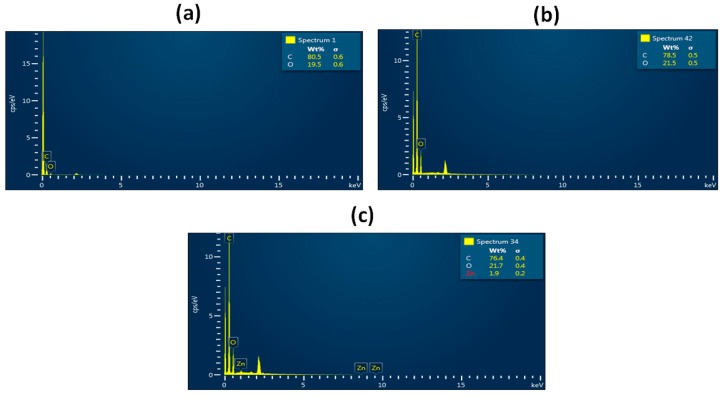
Energy Dispersive X-Ray Spectroscopy (EDX) of (**a**) PU, (**b**) PU/YY and (**c**) PU/YY/ZnNO_3._

Figure 1 shows the SEM images of PU and PU nanocomposite fibers prepared using the electrospinning technique. The SEM images depicted that the developed scaffold consists of smooth nanofibers, randomly oriented without any beads. The fiber diameter was calculated using Image J, and the average fiber diameter of PU, PU/YY and PU/YY/ZnNO_3_ were found to be 869 ± 122 nm, 467 ± 132 nm and 290 ± 163 nm respectively. The fiber diameter analysis showed that the fiber diameter was reduced with the addition of YY and ZnNO_3_. Furthermore, we observed a synergistic reduction of the fiber diameter with the addition of ZnNO_3_ into the polyurethane matrix. The addition of YY resulted in a reduced fiber diameter due to the active constituents present in the oil. This may have been further pronounced when ZnNO_3_ was added to the polyurethane matrix. From the FESEM figure, the ZnNO_3_ particle present on the surface of the fiber was evident, and there might be the possibility of the release of the ZnNO_3_ particle from the fiber surface. However, owing to the low concentration used, there might be no risk of toxicity to the human cells, as indicated in our MTS assay. Few studies have reported that the electrospun scaffold with a smaller diameter facilitated the enhanced osteoblast growth [24,25]. Our developed composites showed a smaller fiber diameter than the pristine PU, suggesting its suitability for the new bone tissue formation. Cell colonization is considered an important parameter in the biological process, and it influences wound healing, vascularization, tissue engineering and stem cell differentiation. The scaffolds are used to support the ingrowth of cells during colonization [26]. Noriega et al. electrospun a chitosan scaffold and studied the chondrocytes’ spreading, proliferation and differentiation. It has been shown that a smaller fiber diameter shows a higher deposition ratio of collagen II/collagen I compared to the lager fiber diameter [27]. In another study, Guo et al. developed scaffolds with fiber diameters of 0.347 μm, 0.947 μm, and 6.48 μm to examine the proliferation and differentiation of osteoblast cells. It was reported that the scaffold with 0.35 μm exhibited a higher projected area and increased the expression of Runt-related transcription factor 2 (RUNX2), type I collagen (Col I), alkaline phosphatase (ALP) activity and osteocalcin (OCN), respectively [28]. Hence, the specific surface area of a smaller fiber diameter facilitated the enhanced protein adhesion, causing a large amount of cell attachment on them [29]. In this work, fabricated composites exhibited a reduced fiber diameter compared to the control, which should be favourable to the cell colonization to induce tissue growth. The presence of zinc (1.9%) in the polyurethane matrix was confirmed in the EDX study, as shown in Figure 2.

### 3.2. IR Analysis

**Figure 3:**
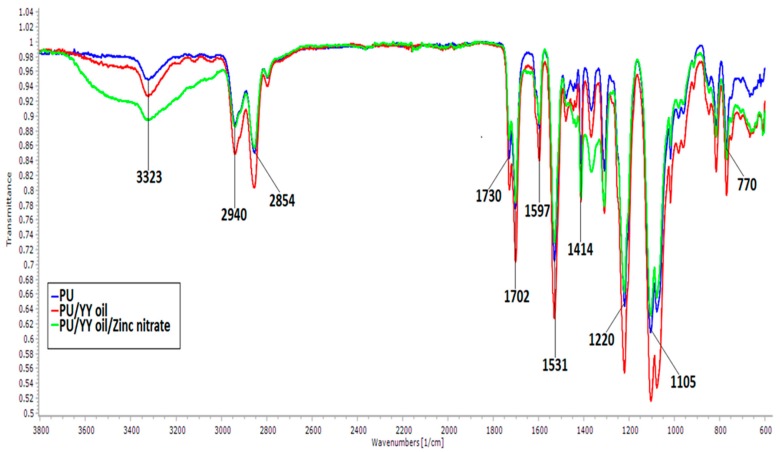
Fourier Transform Infrared Spectroscopy (FTIR) of PU, PU/YY and PU/YY/ZnNO_3._

The IR spectra of the nanofibrous membranes were carried out to examine the functional groups that were present. Figure 3 shows the FTIR spectra of the PU, PU/YY, PU/YY/ZnNO_3_ composite membranes. The pristine PU showed characteristic broad bands of NH stretch at 3323 cm^−1^, and its vibrations were observed at 1531 cm^−1^ and 1597 cm^−1^, respectively. The bands of the CH group can be observed at 2940 cm^−1^ and 2854 cm^−1^, and the band at 1414 cm^−1^ represents its vibrations. Furthermore, a twin peak is seen at 1702 cm^−1^ and 1730 cm^−1^, attributed to the bands of the CO group. Moreover, the characteristic peak was present between the regions of 500–1200 cm^−1^. The bands at 1220 cm^−1^, 1105 cm^−1^ and 770 cm^−1^ denote the characteristic peaks of PU, which are attributed to C–O–C and C–OH stretching vibrations, respectively [17,18,19]. In the electrospun nanocomposites, the peaks were similar to those of the pristine PU. However, an increase in the intensity was observed in the pristine PU while incorporating YY and ZnNO_3_ respectively. The alteration in the peak intensity was due to a strong hydrogen bond formation between the molecules of YY and ZnNO_3_ and the molecules of PU [30].

### 3.3. Contact Angle Measurements

The contact angles measurement was performed to evaluate the wettability and adhesion properties of the developed as-spun membranes. The PU, PU/YY, PU/YY/ZnNO_3_ membranes contact angle were observed to be 106 ± 3°, 112 ± 1°, and 86 ± 1°, respectively. It was shown that the contact angle was increased to 112° while adding YY, indicating hydrophobic behaviour, and that it was decreased to 86° upon adding ZnNO_3_, suggesting hydrophilic behaviour. Hence, the addition of ZnNO_3_ improved the wettability of the pristine PU. The wettability design of a scaffold is important in tissue engineering, as it can influence the cell adhesion and proliferation to a higher extent. It was reported that the contact angle in the range of less than 106° was inappropriate for optimum cell adhesion and proliferation [31]. The contact angle of PU/YY (112° ± 1°) was found to be behind the reported range, which may cause a reduction of the cell adhesion and proliferation. Furthermore, adding ZnNO_3_ to the PU/YY facilitates the improved wettability (86° ± 1°), which lies in the reported wettability range and is suitable for the improved cell adhesion and proliferation for new tissue growth. Liang et al. electrospun a bone scaffold utilizing PLGA/nHA/graphene oxide. It was found that the developed PLGA/HA/graphene oxide scaffolds displayed hydrophilic behaviour and favoured an enhanced osteoblast cell response [32]. Hence, our optimal wettability of the fabricated PU/YY/ZnNO_3_ nanocomposite might serve as a potential candidate for bone tissue engineering.

### 3.4. XRD Analysis

**Figure 4:**
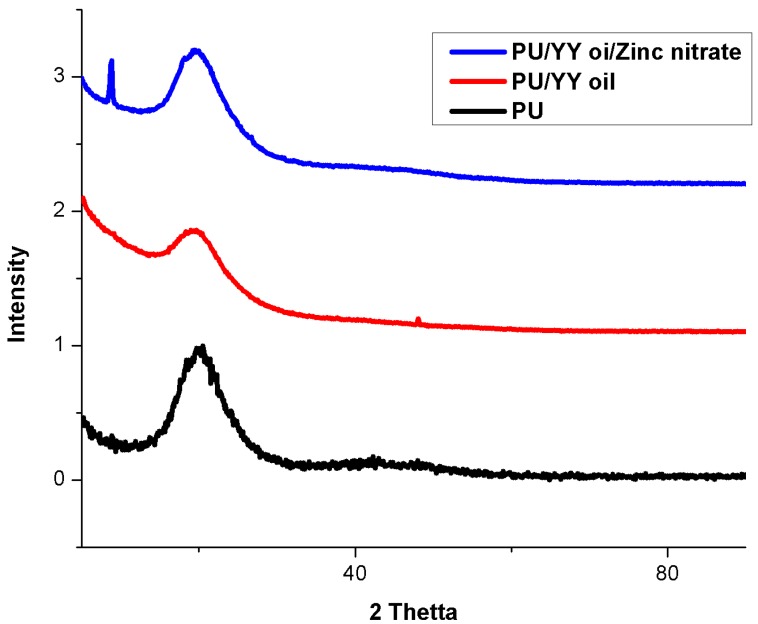
X-ray Diffraction (XRD) images of PU, PU/YY and PU/YY/ZnNO_3._

Figure 4 shows the XRD patterns of the pure PU, PU/YY and PU/YY ZnNO_3_ nanofibers. The pristine PU showed a characteristic broad peak at 20°, which indicates its amorphous behaviour. For the electrospun PU/YY scaffolds, there was no new peak found, apart from the characteristic broad peak at 20°. In the case of PU/YY/ZnNO_3_, there was an extra peak at 8.8°, apart from the broad peak. The peaks were attributed to the crystal plane of Zn [33]. Hence, the XRD analysis confirms the change in crystalline behaviour of the PU/YY/ZnNO_3_ nanocomposites.

### 3.5. Thermal Analysis

**Figure 5:**
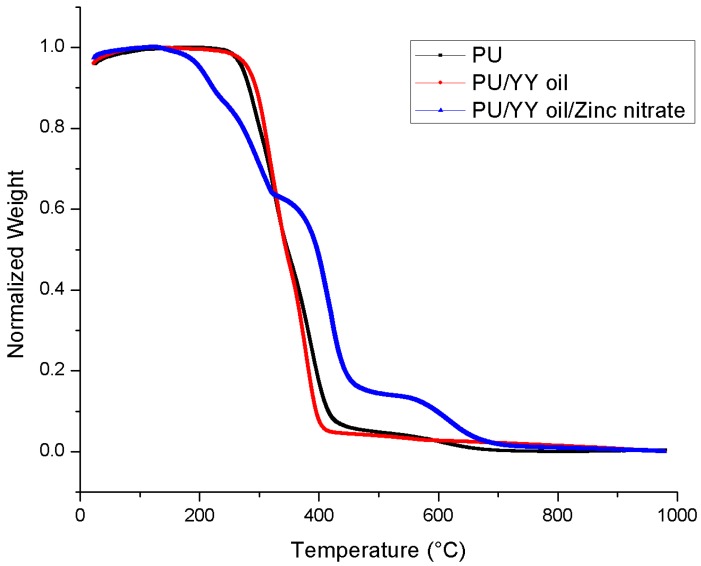
Thermal gravimetric analysis (TGA) of PU, PU/YY and PU/YY/ZnNO_3._

**Figure 6:**
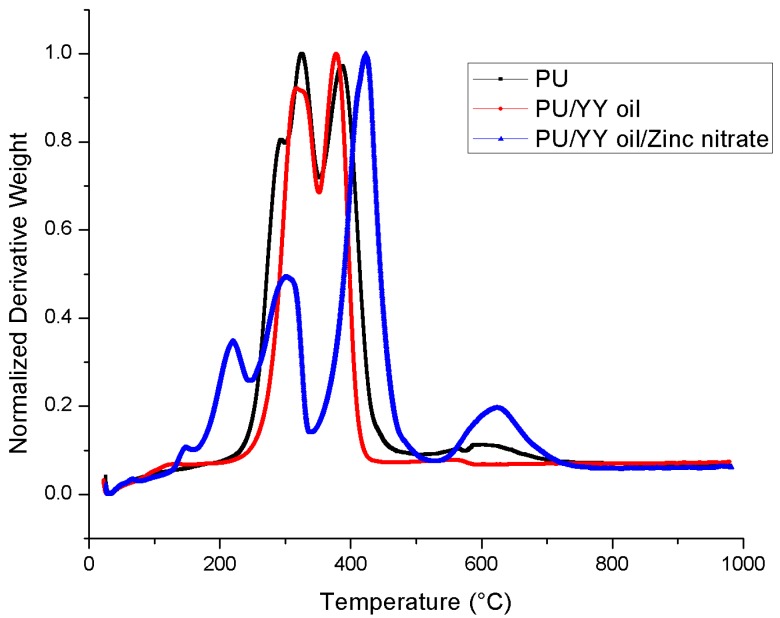
Weight residue of PU, PU/YY and PU/YY/ZnNO_3._

The thermal behaviours of PU, PU/YY, and PU/YY/ZnNO_3_ were done using TGA, and their respective curves are presented in Figure 5. The results for the thermal analysis depicted that the decomposition temperature of the developed PU/YY nanocomposite is much higher than that of the neat PU fiber, while PU/YY/ZnNO_3_ shows lower decomposition temperature than the pristine PU. The pristine PU exhibited an initial decomposition temperature of 266 °C, and for electrospun PU/YY and PU/YY/ZnNO_3_ it was observed to be 273 °C and 196 °C, respectively. Hence, the reinforcement of YY improved the thermal stability of the pristine PU, while the addition of ZnNO_3_ decreased the onset degradation. Jaganathan et al. electrospun polyurethane scaffolds added with zinc nitrate. It was found that the addition of zinc nitrate resulted in a decrease in the onset degradation temperature of PU, which resembles our findings. Furthermore, they attributed this to the evaporation of water or moisture present in the developed film [34]. Further weight loss curves for the fabricated PU, PU/YY, and PU/YY/ZnNO_3_ are indicated in Figure 6, and a list of weight loss peaks are listed in Table 1. The pristine PU showed 4 weight loss peaks, whereas the electrospun PU/YY showed only 2 weight loss peaks, indicating a reduced weight loss compared to the pristine PU. However, with the ZnNO_3_ addition, the number of weight loss peaks increased (5 loss peaks) compared to the pristine PU. This clearly depicted that the added constituents, like YY and ZnNO_3_, are integrated within the polyurethane matrix. A decrease in the intensity of the weight loss peak in electrospun PU/YY/ZnNO_3_ compared to the pristine PU indicated the reduced weight loss of their fabricated composites. This is quite similar to recent observation where Jaganathan et al. fabricated polyurethane scaffolds added with zinc nitrate. It was observed that the addition of zinc nitrate resulted in a decrease in the weight loss peak intensity of PU, which resembles our findings [34].

### 3.6. Mechanical Properties

**Figure 7:**
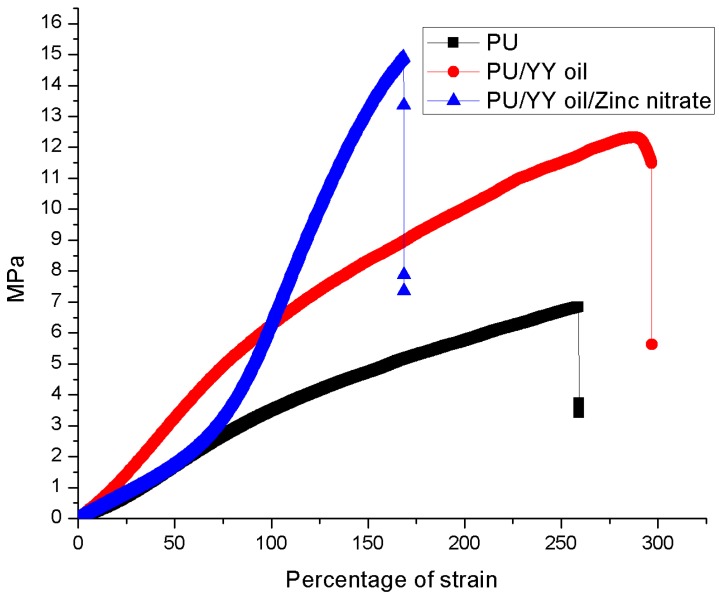
Tensile strength of PU, PU/YY and PU/YY/ZnNO_3._

The mechanical properties of the PU, PU/YY and PU/YY/ZnNO_3_ nanocomposites were determined using a tensile test, and their corresponding stress-strain plots are depicted in Figure 7. It was observed that the ultimate tensile strength of YY and ZnNO_3_ incorporated nanofibers were higher than pure nanofibers. The tensile strength of the pristine PU was found to be 6.83 MPa, whereas PU incorporated with YY and YY/ZnNO_3_ showed a strength of 12.32 MPa and 14.90 MPa respectively. Hence, the addition of YY and ZnNO_3_ significantly enhanced the mechanical performance of the pristine PU. Jaganathan et al. developed an electrospun scaffold utilizing polyurethane and copper sulphate. It was found that the incorporation of copper sulphate resulted in the enhancement of the mechanical strength, which correlates with our findings. They reported that this improved mechanical performance resulted from their smaller fiber diameter [35]. Hence, the developed composites showed a smaller fiber diameter than the pristine, which resulted in the improvement of the tensile strength. It has been reported that the mechanical strength in the range between 4 to 10 MPa is suitable for human fetal osteoblast cells response [36]. In the human body, the osteoblast was largely present in the periosteum, which is the thin connective tissue layer that lies outside the surface of bones and in the endosteum [37]. In this research, the reported mechanical strength matches those reported values, indicating its potentiality for periosteum tissue growth.

### 3.7. AFM Analysis

**Figure 8:**
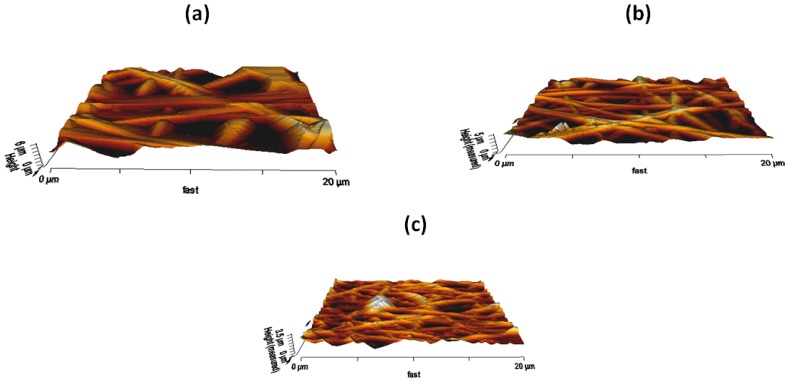
Atomic force microscopy (AFM) images of (**a**) PU, (**b**) PU/YY and (**c**) PU/YY/ZnNO_3._

Additionally, the surface morphologies of PU, PU/YY, PU/YY/ZnNO_3_ nanocomposite fibers were evaluated by atomic force microscopy. The corresponding AFM images of the as-spun nanofibers are shown in Figure 8. The average surface roughness of the PU was reported to be 776 nm, and the surface roughness of the PU incorporated with YY and ZnNO_3_ was found to be 524 nm and 284 nm, respectively. The YY and ZnNO_3_ addition resulted in the reduction of the roughness of the PU, indicating that its surfaces were smooth. Kim et al. investigated the effect of the fiber diameter on the surface roughness of electrospun Poly(ε–caprolactone) scaffolds. It had been found that a scaffold with a smaller fiber diameter showed a smoother morphology and became rougher when the diameter became larger [38]. Hence, our fabricated composites with a smaller fiber diameter might have facilitated the smoother surfaces. Ribeiro developed poly (L–lactide) membranes and investigated the osteoblast cells proliferation. It was found that the surfaces having a low surface roughness displayed an increased adhesion and proliferation of osteoblast cells, compared to the surfaces having a higher surface roughness [39]. Hence, in our study, the lower surface roughness of the developed nanocomposites might be suitable for the enhanced osteoblast cell response.

### 3.8. Coagulation Assessments

**Figure 9:**
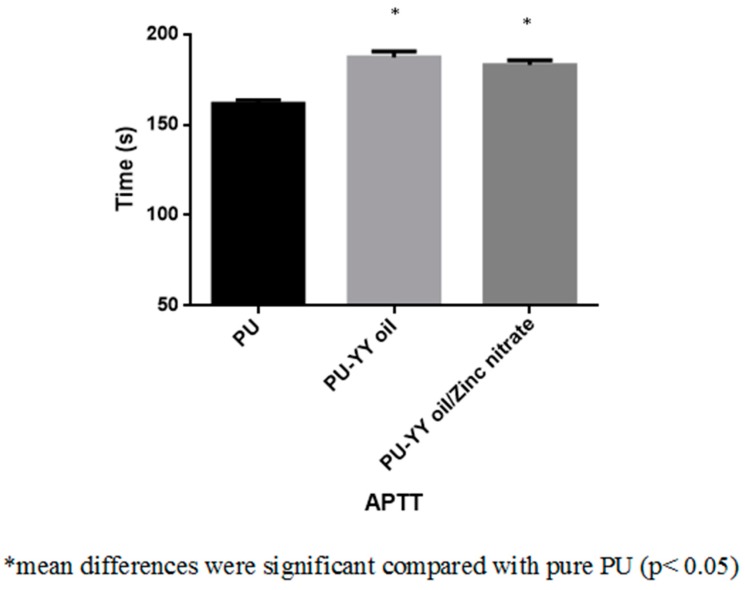
Activated Partial Thromboplastin Time (APTT) assay of PU, PU/YY and PU/YY/ZnNO_3._

**Figure 10:**
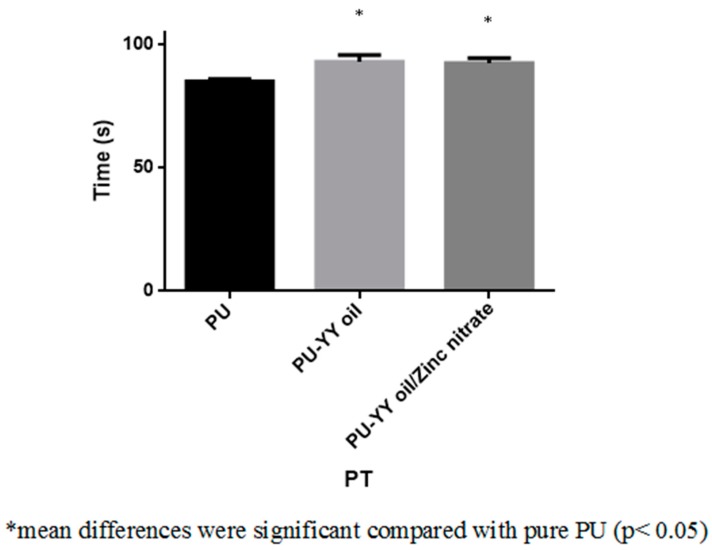
Prothrombin Time (PT) assay of PU, PU/YY and PU/YY/ZnNO_3._

**Figure 11:**
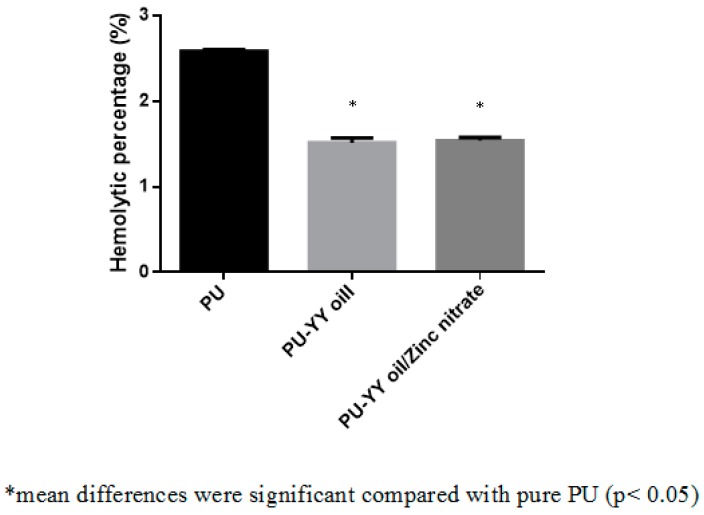
Hemolytic assay of PU, PU/YY and PU/YY/ZnNO_3._

The coagulation assay assessments for the electrospun PU, PU/YY and PU/YY/ZnNO_3_ scaffold are presented in Figure 9, Figure 10 and Figure 11. The APTT time for the electrospun PU/YY and PU/YY/ZnNO_3_ scaffold was reported to be 187 ± 3 s and 183 ± 3 s, respectively, whereas for the pristine PU it was 162 ± 2 s. Similarly, The PT time for the electrospun PU/YY and PU/YY/ZnNO_3_ scaffold was reported to be 93 ± 3 s and 92 ± 2 s, respectively, whereas for the pristine PU it was 85 ± 1 s. The APTT and PT measurements clearly depicted that the developed nanocomposites showed a better compatibility with blood, signified by a prolonging of the blood clotting times when compared to pure polyurethane. This might be due to the presence of YY and ZnNO_3_ in the polyurethane matrix. Furthermore, a hemolytic percentage was also performed, in which the pristine PU showed a hemolytic percentage of 2.58%, while for the electrospun PU/YY and PU/YY/ZnNO_3_ scaffold the hemolytic percentages were 1.51% and 1.54%, respectively, indicating non-hemolytic materials, according to ASTMF756-00 (2000) [18]. The blood compatibility measurements depicting a significant delay in blood clotting for the electrospun PU/YY nanocomposites might result from its hydrophobic behaviour. In the blood/surface interaction, the hydrophobic surface will favour the irreversible adhesion of plasma proteins, which results in a delay in the blood clotting time. However, there was variance in the blood clotting times when adding ZnNO_3_ to the PU/YY nanocomposites. This variance was due to the change in the wettability behaviour of the PU/YY/ZnNO_3_ nanocomposites [40]. Jaganathan electrospun polyurethane scaffolds added with corn and neem oil. It was observed that the addition of corn and neem oil resulted in enhanced blood clotting times compared to the pristine PU, and the researchers concluded that this might be due to the smaller fiber diameter and hydrophobic nature of the fabricated composites [18]. In this research, the smaller fiber diameter (PU/YY and PU/YY/ZnNO_3_) and hydrophobic behaviour (PU/YY) of the electrospun nanocomposites might play a putative role in enhancing the anticoagulant behaviour of the pristine polyurethane.

### 3.9. Bone Mineralization Testing

**Figure 12:**
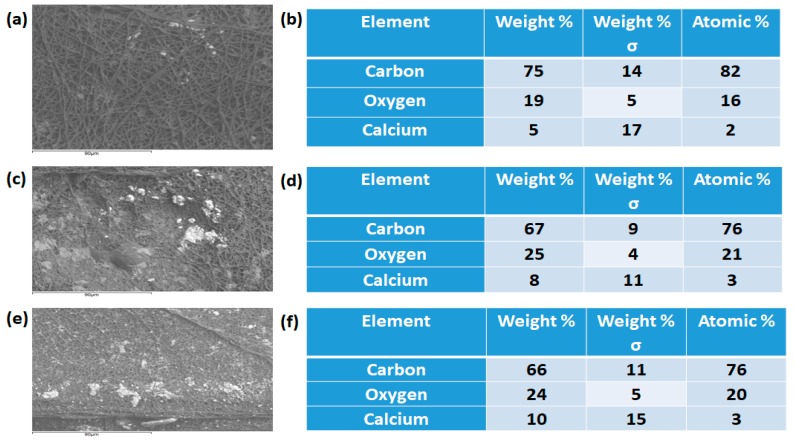
Scanning electron microscopy (SEM) and Energy Dispersive X-Ray Spectroscopy (EDX) of calcium deposition of (**a**,**b**) PU, (**c**,**d**) PU/YY and (**e**,**f**) PU/YY/ZnNO_3._

Figure 12 presents a representation diagram describing the bone mineralization process of the electrospun membranes in SBF after 14 days. It was observed that the presence of YY and zinc nitrate accelerates the calcium deposition in the pristine PU. The weight percentage of calcium deposited in the pristine PU was found to be 5.323%, and for electrospun PU/YY and PU/YY/ZnNO_3_ it was reported to be 7.919% and 10.150%, respectively. Hence, the fabricated composites with an improved calcium deposition might serve as potential candidates for bone tissue engineering.

### 3.10. Cytocompatibility Analysis

**Figure 13:**
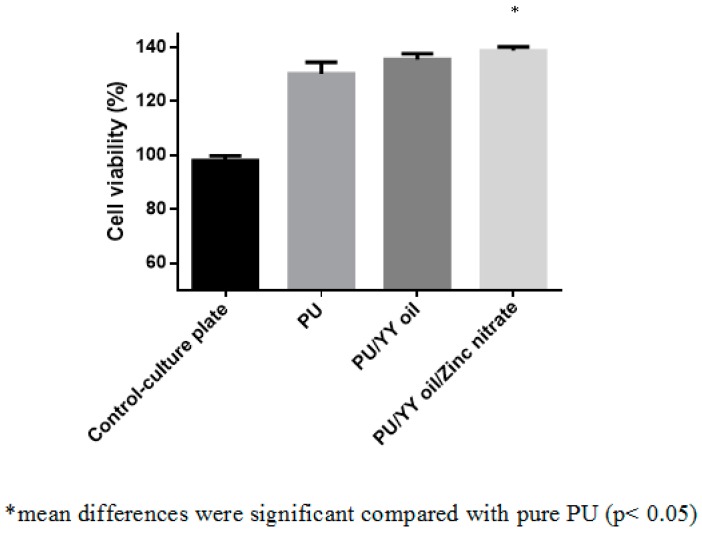
Cell viability of PU, PU/YY and PU/YY/ZnNO_3._

The fibroblast cell viabilities of the electrospun PU, PU/YY and PU/YY/ZnNO_3_ nanofibrous scaffold using the MTS assay were presented in Figure 13. After a 5 day culture, the PU membrane showed a cell viability in the range of 130 ± 4%, and for the electrospun PU/YY and PU/YY/ZnNO_3_ scaffold it showed a viability of 136 ± 2% and 139 ± 1%, respectively. It was shown that all electrospun membranes showed a better cell viability than the control. Furthermore, the cell viability of the electrospun PU/YY and PU/YY/ZnNO_3_ nanofibers were found to be higher than the pristine PU. As reported earlier, the smaller fiber diameter and hydrophilic behaviour might favour the fibroblast cell adhesion and proliferation. This enhanced cell adhesion of the fibroblast may be due to the reduced fiber diameter (PU/YY and PU/YY/ZnNO_3_) and hydrophilic behaviour (PU/YY and PU/YY/ZnNO_3_) of the developed composites [41,42]. The periosteum in the human bone is divided into two layers, namely the outer layer which is fibrous and the inner layer which is cambium. The fibrous layer is composed of fibroblasts, while the cambium layer has progenitor cells that grow into osteoblasts [43]. Since our fabricated composites showed a non-toxic behaviour to the fibroblast cells, this indicated the suitability for the growth of periosteum tissue.

## 4. Conclusions

This research successfully developed a novel scaffold utilizing PU nanofibers added with ylang ylang (YY) and zinc nitrate (ZnNO_3_) using the electrospinning method. The SEM images showed that the diameter of the PU nanofibers was reduced with the addition of YY and ZnNO_3_. FTIR, TGA and XRD analyses confirmed the interactions between PU with YY and ZnNO_3._ The TGA study revealed the improved thermal stability for PU/YY and a slight reduction in the thermal stability for PU/YY/ZnNO_3._ The tensile test indicated that the addition of YY and ZnNO_3_ improved the mechanical properties of the electrospun nanofibers. The electrospun PU/YY and PU/YY/ZnNO_3_ showed a reduced surface roughness when compared with the pristine PU, as depicted in the AFM analysis. The addition of YY and ZnNO_3_ improved the anticoagulant and biocompatibility nature of the pristine PU, as depicted in the coagulation and MTS assays. Furthermore, the bone mineralization study depicted the improved calcium deposition in the fabricated composites, when compared with the pristine PU. Hence, the developed composites showed desirable physico-chemical properties, biocompatibility and calcium deposition, and might be plausible candidates for bone tissue engineering. However, a thorough in-vivo investigation on the biodegradation and regeneration of the fabricated membranes should be evaluated for further approval.

## Figures and Tables

**Table 1:** Weight loss peaks of the electrospun membranes.

S.NO	Polyurethane (PU)	PU/Ylang Ylang (YY)	PU/YY/Zinc Nitrate (ZnNO_3_)
First weight loss	210 °C to 302 °C	225 °C to 351 °C	119 °C to 161 °C
Second weight loss	302 °C to 353 °C	351 °C to 470 °C	161 °C to 246 °C
Third weight loss	353 °C to 494 °C	-	246 °C to 339 °C
Fourth weight loss	494 °C to 760 °C	-	339 °C to 530 °C
Fifth weight loss	-	-	530 °C to 780 °C

## References

[1] Qi J., Zhang H., Wang Y., Mani M.P., Jaganathan S.K. (2018). Development and blood compatibility assessment of electrospun polyvinyl alcohol blended with metallocene polyethylene and plectranthus amboinicus (PVA/mPE/PA) for bone tissue engineering. Int. J. Nanomed..

[2] Bone Regeneration. https://www.sciencedirect.com/topics/medicine-and-dentistry/bone-regeneration.

[3] Son S.R., Linh N.T., Yang H.M., Lee B.T. (2013). In vitro and in vivo evaluation of electrospun PCL/PMMA fibrous scaffolds for bone regeneration. Sci. Technol. Adv. Mater..

[4] Thavornyutikarn B., Chantarapanich N., Sitthiseripratip K., Thouas G.A., Chen Q. (2014). Bone tissue engineering scaffolding: Computer-aided scaffolding techniques. Prog. Biomater..

[5] Chan B.P., Leong K.W. (2008). Scaffolding in tissue engineering: General approaches and tissue-specific considerations. Eur. Spine J..

[6] Sill T.J., Von Recum H.A. (2008). Electrospinning: Applications in drug delivery and tissue engineering. Biomaterials.

[7] Voytik-Harbin S.L. (2001). Chapter 26 Three-dimensional extracellular matrix substrates for cell culture. Methods Cell Biol..

[8] Bhattarai R., Bachu R., Boddu S., Bhaduri S. (2019). Biomedical applications of electrospun nanofibers: Drug and nanoparticle delivery. Pharmaceutics.

[9] Subbiah T., Bhat G.S., Tock R.W., Parameswaran S., Ramkumar S.S. (2005). Electrospinning of nanofibers. J. Appl. Polym. Sci..

[10] Huang Z.-M., Zhang Y.-Z., Kotaki M., Ramakrishna S., Zhang Y. (2003). A review on polymer nanofibers by electrospinning and their applications in nanocomposites. Compos. Sci. Technol..

[11] Wu S.-C., Chang W.-H., Dong G.-C., Chen K.-Y., Chen Y.-S., Yao C.-H. (2011). Cell adhesion and proliferation enhancement by gelatin nanofiber scaffolds. J. Bioact. Compat. Polym..

[12] Polymer Properties Database. https://polymerdatabase.com/polymer%20classes/Polyurethane%20type.html.

[13] Shen Z., Lu D., Li Q., Zhang Z., Zhu Y. (2015). Synthesis and Characterization of Biodegradable Polyurethane for Hypopharyngeal Tissue Engineering. BioMed Res. Int..

[14] Kumar N.S., Santhosh C., Sudakaran S.V., Deb A., Raghavan V., Venugopal V., Bhatnagar A., Bhat S., Andrews N.G. (2017). Electrospun polyurethane and soy protein nanofibres for wound dressing applications. IET Nanobiotechnol..

[15] Gencturk A., Kahraman E., Güngör S., Özhan G., Özsoy Y., Sarac A.S. (2017). Polyurethane/hydroxypropyl cellulose electrospun nanofiber mats as potential transdermal drug delivery system: Characterization studies and in vitro assays. Artif. Cells Nanomed. Biotechnol..

[16] Gabriel L.P., Rodrigues A.A., Macedo M., Jardini A.L., Filho R.M. (2017). Electrospun polyurethane membranes for Tissue Engineering applications. Mater. Sci. Eng. C.

[17] Jaganathan S.K., Mani M.P., Nageswaran G., Krishnasamy N.P., Ayyar M. (2018). Single stage electrospun multicomponent scaffold for bone tissue engineering application. Polym. Test..

[18] Jaganathan S.K., Mani M.P., Palaniappan S.K., Rathanasamy R. (2018). Fabrication and characterisation of nanofibrous polyurethane scaffold incorporated with corn and neem oil using single stage electrospinning technique for bone tissue engineering applications. J. Polym. Res..

[19] Chao C.Y., Mani M.P., Jaganathan S.K. (2018). Engineering electrospun multicomponent polyurethane scaffolding platform comprising grapeseed oil and honey/propolis for bone tissue regeneration. PLoS ONE.

[20] De Silva R.T., Mantilaka M.M.M.G.P.G., Goh K.L., Ratnayake S.P., Amaratunga G.A.J., De Silva K.M.N. (2017). Magnesium Oxide Nanoparticles Reinforced Electrospun Alginate-Based Nanofibrous Scaffolds with Improved Physical Properties. Int. J. Biomater..

[21] Rodriguez-Tobias H., Morales G., Ledezma A., Romero J., Grande D. (2014). Novel antibacterial electrospun mats based on poly(d,l-lactide) nanofibers and zinc oxide nanoparticles. J. Mater. Sci..

[22] Tan L.T.H., Lee L.H., Yin W.F., Chan C.K., Kadir H.A., Chan K.G., Goh B.H. (2015). Traditional Uses, Phytochemistry, and Bioactivities of Cananga odorata (Ylang-Ylang). Evidence-Based Complement. Altern. Med..

[23] Forero J.C., Roa E., Reyes J.G., Acevedo C., Osses N. (2017). Development of Useful Biomaterial for Bone Tissue Engineering by Incorporating Nano-Copper-Zinc Alloy (nCuZn) in Chitosan/Gelatin/Nano-Hydroxyapatite (Ch/G/nHAp) Scaffold. Materials.

[24] Linh N.T., Lee B.T. (2012). Electrospinning of polyvinyl alcohol/gelatin nanofiber composites and cross-linking for bone tissue engineering application. J. Biomater. Appl..

[25] Prabhakaran M.P., Venugopal J., Ramakrishna S. (2009). Electrospun nanostructured scaffolds for bone tissue engineering. Acta Biomater..

[26] Lawrence B.J., Madihally S.V. (2008). Cell colonization in degradable 3D porous matrices. Cell Adhes. Migr..

[27] Noriega S.E., Hasanova G.I., Schneider M.J., Larsen G.F., Subramanian A. (2012). Effect of fiber diameter on the spreading, proliferation and differentiation of chondrocytes on electrospun chitosan matrices. Cells Tissues Organs.

[28] Guo Z., Ma M., Huang X., Li H., Zhou C. (2017). Effect of Fiber Diameter on Proliferation and Differentiation of MC3T3-E1 Pre-Osteoblasts. J. Biomater. Tissue Eng..

[29] Mani M.P., Jaganathan S.K. (2019). Physicochemical and blood compatibility characteristics of garlic incorporated polyurethane nanofibrous scaffold for wound dressing applications. J. Text. Inst..

[30] Unnithan A.R., Pichiah P.T., Gnanasekaran G., Seenivasan K., Barakat N.A., Cha Y.-S., Jung C.-H., Shanmugam A., Kim H.Y. (2012). Emu oil-based electrospun nanofibrous scaffolds for wound skin tissue engineering. Colloids Surfaces A Physicochem. Eng. Asp..

[31] Wei J., Igarashi T., Okumori N., Igarashi T., Maetani T., Liu B., Yoshinari M. (2009). Influence of surface wettability on competitive protein adsorption and initial attachment of osteoblasts. Biomed. Mater..

[32] Liang C., Luo Y., Yang G., Xia D., Liu L., Zhang X., Wang H. (2018). Graphene Oxide Hybridized nHAC/PLGA Scaffolds Facilitate the Proliferation of MC3T3-E1 Cells. Nanoscale Res. Lett..

[33] Zhang Y., Zhu F., Zhang J., Xia L. (2008). Converting Layered Zinc Acetate Nanobelts to One-dimensional Structured ZnO Nanoparticle Aggregates and their Photocatalytic Activity. Nanoscale Res. Lett..

[34] Jaganathan S.K., Mani M.P. (2019). Single-stage synthesis of electrospun polyurethane scaffold impregnated with zinc nitrate nanofibers for wound healing applications. J. Appl. Polym. Sci..

[35] Jaganathan S.K., Mani M.P. (2018). Electrospun polyurethane nanofibrous composite impregnated with metallic copper for wound-healing application. 3 Biotech.

[36] Salifu A.A., Lekakou C., Labeed F.H. (2017). Electrospun oriented gelatin-hydroxyapatite fiber scaffolds for bone tissue engineering. J. Biomed. Mater. Res. Part. A.

[37] Anatomy and Physiology. https://opentextbc.ca/anatomyandphysiology/chapter/6-3-bone-structure/.

[38] Kim H.H., Kim M.J., Ryu S.J., Ki C.S., Park Y.H. (2016). Effect of fiber diameter on surface morphology, mechanical property, and cell behavior of electrospun poly (ε-caprolactone) mat. Fiber Polym..

[39] Ribeiro C., Sencadas V., Areias A.C., Gama F.M., Lanceros-Méndez S. (2015). Surface roughness dependent osteoblast and fibroblast response on poly (l-lactide) films and electrospun membranes. J. Biomed. Mater. Res. Part. A.

[40] Szycher M. (2017). High Performance Biomaterials: A Complete Guide to Medical and Pharmceutical Applications.

[41] Tian F., Hosseinkhani H., Hosseinkhani M., Khademhosseini A., Yokoyama Y., Estrada G.G., Kobayashi H. (2008). Quantitative analysis of cell adhesion on aligned micro-and nanofibers. J. Biomed. Mater. Res. Part. A.

[42] Nandakumar V., Suresh G., Chittaranjan S., Doble M. (2012). Synthesis and Characterization of Hydrophilic High Glycolic Acid–Poly(d,l-Lactic-co-Glycolic Acid)/Polycaprolactam/Polyvinyl Alcohol Blends and Their Biomedical Application as a Ureteral Material. Ind. Eng. Chem. Res..

[43] Periosteum. https://en.wikipedia.org/wiki/Periosteum.

